# Association Between Sub-National Regional Socioeconomic Status and Childhood Obesity in Five South-East European Countries: The WHO European Childhood Obesity Surveillance Initiative—COSI (2019)

**DOI:** 10.3390/children13020267

**Published:** 2026-02-13

**Authors:** Sanja Musić Milanović, Helena Križan, Nika Šlaus, Emanuel Brađašević, Maja Lang Morović, Visnja Djordjic, Enisa Kujundžić, Sergej M. Ostojic, Igor Spiroski, Gregor Starc

**Affiliations:** 1 Croatian Institute of Public Health, 10000 Zagreb, Croatia; sanja.music@hzjz.hr (S.M.M.); slausnik@gmail.com (N.Š.); emanuel.bradasevic@hzjz.hr (E.B.); maja.lang-morovic@hzjz.hr (M.L.M.); 2 School of Public Health “Andrija Štampar”, University of Zagreb School of Medicine, 10000 Zagreb, Croatia; 3 ECDC Fellowship Programme, Field Epidemiology Path (EPIET), European Centre for Disease Prevention and Control (ECDC), 171 83 Stockholm, Sweden; 4 Faculty of Sport and Physical Education, University of Novi Sad, 21000 Novi Sad, Serbia; visnja@uns.ac.rs (V.D.); sergej.ostojic@uia.no (S.M.O.); 5 Institute of Public Health of Montenegro, 81000 Podgorica, Montenegro; enisa.kujundzic@ijzcg.me; 6 Department of Nutrition and Public Health, University of Agder, 4604 Kristiansand, Norway; 7 Faculty of Health Sciences, University of Pecs, 7622 Pecs, Hungary; 8 Institute for Public Health of the Republic of N. Macedonia, 1000 Skopje, North Macedonia; i.spiroski@iph.mk; 9 Faculty of Medicine, Ss. Cyril and Methodius University, 1000 Skopje, North Macedonia; 10 Faculty of Sport, University of Ljubljana, 1000 Ljubljana, Slovenia; gregor.starc@fsp.uni-lj.si

**Keywords:** childhood obesity, sub-national, regional, socioeconomic, south-east Europe

## Abstract

Background/Objectives: This study focused on the sub-national regional heterogeneity in childhood obesity prevalence across five countries in south-east Europe and the correlation between this heterogeneity and socioeconomic differences. Previous studies have mainly observed national or cross-national data but this study used a sub-national regional approach that may be beneficial in the further investigation of childhood obesity. Methods: Nationally representative samples of children from Croatia, Montenegro, North Macedonia, Serbia and Slovenia were selected using the COSI methodology and used to estimate regional childhood obesity prevalence values. The Sub-national Human Development Database provided data on the Sub-national Human Development Index (SHDI). The spatial autocorrelation analysis of childhood obesity prevalence in sub-national regions was performed and its association with sub-national human development was tested with an ordinary least squares regression model. Results: This study found statistically significant differences in childhood obesity prevalence across sub-national regions in Croatia, Slovenia and Serbia, while no such differences were observed in North Macedonia and Montenegro. There was moderate clustering in childhood obesity rates (Moran’s I = 0.337). The results indicated a significant negative association between SHDI and childhood obesity prevalence across the 48 regions (β = −66.63, *p* < 0.001). Conclusions: Future public health efforts should take into consideration regional differences in childhood obesity prevalence, and more targeted research is essential for understanding the mechanisms of resilience and vulnerability on a sub-national level.

## 1. Introduction

A continuous rise in childhood obesity presents a global threat to public health [1,2,3]. The COVID-19 pandemic has further highlighted and confirmed the detrimental consequences of obesity on health [4]. Hence, it is now more critical than ever to develop effective interventions and expand our knowledge and understanding of the mechanisms underlying childhood obesity.

To collect comparable data on childhood overweight and obesity metrics in a standardized way with measured anthropometric values, the World Health Organization (WHO) Regional Office for Europe initiated the WHO European Childhood Obesity Surveillance Initiative (COSI). The first round of COSI was conducted in 2007, with the most recent round taking place between 2021 and 2023. In the most recent round, data on approximately 470,000 schoolchildren were gathered, creating a robust evidence base to guide the development of policies and strategies for childhood obesity prevention [2].

The results from the fifth round of the COSI research, implemented between 2018 and 2020, reveal that the highest prevalence of childhood obesity, in both boys and girls, has been observed in Mediterranean countries. Among boys, the highest rates were recorded in Cyprus (24.1%), Greece (23.1%), and Italy (19.8%), while among girls Cyprus (15.3%), Spain (14.8%) and Italy (12.9%) reported the highest prevalence. Conversely, the lowest prevalence was found in Denmark, with rates of 6.6% in boys and 4.8% in girls [3]. In the south-east European region, the prevalence of childhood obesity ranges from 19.7% in Cyprus to 9.6% in Slovenia. Additionally, differences in childhood obesity rates have been observed within countries, at regional and sub-regional levels [5,6,7,8,9,10]. These findings underscore the potential limitations of using national reference values to define childhood obesity.

Many studies exploring variations in childhood obesity focus on smaller spatial units, such as neighborhoods, and examine characteristics in relation to the prevalence of obesity among children living and attending schools in those areas. These characteristics often include the availability of green spaces [11,12], the density of fast-food restaurants [13,14], and the presence of food retailers [15]. Socioeconomic factors play a crucial role, as research indicates that obesity rates are generally lower in affluent neighborhoods than in disadvantaged ones [14,16,17].

A substantial body of research underscores the link between socioeconomic status and childhood obesity [18,19]. To gain deeper insights into socioeconomic variables at sub-national, regional, and local levels, the Joint Research Center (JRC) of the European Commission developed the Socioeconomic Regional Microscope—a series of periodic publications designed to stimulate research through regional data, visuals, and narrative analysis [20]. One such JRC report, “Mapping and Zooming in on Childhood Overweight and Obesity”, explored variations in childhood obesity across regional, local, and socioeconomic contexts. Examining childhood obesity at the sub-national regional level is therefore crucial for understanding its origins and for the identification of the underlying factors that influence children’s health [21]. Additionally, this approach can offer valuable public health insights and benefits on regional and national levels. Regional policymakers play a pivotal role in shaping the environments where children spend most of their time—such as schools, sports clubs, parks, and public transportation systems—which significantly impact behavior and are essential for preventing childhood obesity, while national policy makers can shape policies that support regional challenges [11,22,23,24]. Since 1990, the United Nations Development Program (UNDP) has been publishing the Human Development Index (HDI) annually. The HDI is an aggregate measure of achievements in the standard of living, education, and health, which is frequently used to assess wellbeing across countries. However, the HDI focuses solely on the national level, potentially masking disparities across sub-national regions. To address this limitation, the Sub-national Human Development Index (SHDI) was developed. The SHDI is a sub-national version of the HDI that averages sub-national regional values across the three dimensions, education, health, and standard of living [25,26], within which, in our case, meaningful variability is driven primarily by education and income.

This ecological study explores sub-national regional variations in childhood obesity prevalence across five south-east European countries and examines their association with socioeconomic disparities, as measured by the SHDI. While previous multi-national COSI studies have focused solely on national-level data, this study will be the first to offer a detailed analysis at the sub-national regional level.

## 2. Materials and Methods

### 2.1. Study Design and Sampling

The fifth round of data collection in the COSI study, conducted between 2018 and 2020, included five south-east European countries: Croatia, Montenegro, North Macedonia, Serbia, and Slovenia [3]. Nationally representative samples of children were selected in each country, with collaboration from the educational system to facilitate school-based participation. Four of the countries employed a two-stage cluster sampling method, using primary schools as the primary and school classes as the secondary sampling unit. Croatia, however, used a single-stage cluster design with classes as the sampling units. Further details on the implementation and characteristics of the COSI study in 2021–2022 can be found elsewhere [3,27]. All participating countries followed a unified COSI protocol [28,29], which adheres to the International Ethical Guidelines for Biomedical Research Involving Human Subjects [30]. Local ethical committees in each country additionally approved the protocol and methods for their respective national studies. According to the COSI protocol, countries could select one or more of the following age groups: 6.0–6.9, 7.0–7.9, 8.0–8.9, or 9.0–9.9 years. In the fifth round, North Macedonia and Montenegro targeted only 7 year olds, Croatia targeted only 8 year olds, while Slovenia and Serbia included all age groups.

Trained examiners recorded information on children’s weight, height, gender, and age in the Child’s Record Form. Consent was always requested from children immediately before taking anthropometric measurements. In Slovenia, data from the sampled primary schools was collected through the annual national SLOfit measurements, with parental consent. Fieldworkers trained in WHO-standardized techniques measured children’s weight (in grams) and height (in centimeters) [28,29]. Data collection occurred during the defined study period for round five (2018–2020). Depending on the local circumstances and available resources, countries selected suitable professionals—such as physical education teachers or healthcare professionals—to perform the measurements at the national or sub-national regional level.

The inclusion criteria were as follows:

(1) Children aged between 6 and 9 years.
(2) The availability of data on sex, age, sub-national region, height, and weight.

It should be noted that the COSI sampling method is designed to be nationally representative and not specifically to produce representative estimates on a more granular level. Consequently, sub-national prevalence estimates and subsequent spatial analyses should be interpreted with caution. In order to mitigate this limitation, sensitivity analyses were performed to assess the robustness of the spatial patterns and associations.

### 2.2. Children’s Weight Status

Children’s weight status/body mass index (BMI) was classified using the 2007 WHO-recommended growth reference for school-aged children and adolescents [31]. BMI-for-age Z-scores were calculated based on the WHO 2007 cut-offs to estimate the prevalence of childhood obesity. Obesity was defined as a BMI-for-age value > +2 Z-scores. Children with biologically implausible BMI-for-age values (below −5 or above +5 Z-scores relative to the 2007 WHO growth reference median) were excluded from the analysis [32]. While BMI as a measure has limitations derived from not accounting for body composition, it is still the most widely used measure of weight status in population-based studies of children, which is why it was used in this study.

### 2.3. Sub-National Human Development Index and Sub-National Gender Development Index

SHDI data were sourced from the Sub-national Human Development Database [25], which covers 1625 regions across 161 countries from 1990 to 2022. The data from this database has been computed using data from statistical offices and from the Area Database of the Global Data Lab. In this study the SHDI was used, which is derived from the dimensions and indicators outlined in Table 1 [26]. The health index dimension of SHDI in Croatia, Serbia and Montenegro showed no sub-national variation and thus SHDI should be interpreted primarily as a composite indicator reflecting socioeconomic and educational differences.

### 2.4. Statistical Analysis

A macro-level approach was employed, using sub-national region-level data. For each sub-national region included in the analysis, the total prevalence of childhood obesity was estimated.

The differences in childhood obesity between the sub-national regions within specific countries were tested using Pearson’s χ2 test on crude counts per region. All the assumptions for this test were met.

These statistical analyses were conducted using SPSS version 28.0.1.0. A significance level of 5% was applied throughout.

#### Spatial Data Analysis

In order to visualize the data of the included countries with the aggregation level to the regions used for statistical analysis, a map with information about prevalence per region was constructed using QGIS 3.40.11—Bratislava. Spatial analyses were performed in GeoDa 1.22.0.21., followed by additional regression analyses in R (4.3.1) using the sandwich, sf, spdep and lmtest packages.

First, a Queen’s contiguity matrix of the regions that share borders was produced. The matrix was used for calculating univariate Moran’s I coefficient as the measure of global spatial autocorrelation in the dataset. Given the moderate number of spatial units (*n* = 48), the statistical significance was assessed using permutation-based randomization at 99.999 permutations to calculate the pseudo-*p*-value and z-value. The results were visualized with a scatterplot to illustrate the strength and direction of spatial autocorrelation.

The distribution of prevalence values is plotted as a univariate spatial map with boxplot fences (hinge = 3.0) enabling the detection of possible outliers. For cluster analysis, a Local Indicators of Spatial Autocorrelation (LISA) analysis was performed [33]. The clusters are shown on the map as hot-spots of four possible scenarios: (1) “low–low”, (2) “low–high”, (3) “high–high” and (4) “high–low”, enabling the identification of regions which contribute to the overall spatial autocorrelation.

A regression analysis was performed in R where an ordinary least squares (OLS) model was fitted with childhood obesity prevalence as the dependent variable and SHDI as the predictor, with the country included as a covariate to account for the national context. Robust and clustered standard errors were computed to account for heteroskedasticity and within-country correlation. Spatial diagnostics were performed on residuals using MORAN’s I and Rao’s score (LM) test to evaluate spatial lag or error dependence.

Sensitivity analysis was performed by excluding four sub-national regions (all in Croatia) where the number of children included in the study was less than 50, as their estimates were deemed unreliable due to the small number of study participants. All analyses were re-run on this subset of 44 regions.

## 3. Results

This study included 25,211 school-aged children from five south-east European countries: Croatia, Slovenia, North Macedonia, Montenegro and Serbia.

### 3.1. Childhood Obesity Prevalence Across Sub-National Regions

In three of the five countries, there was a statistically significant difference in childhood obesity prevalence among sub-national regions: Croatia (χ^2^ = 56.926; *p* < 0.001), Slovenia (χ^2^ = 66.183; *p* < 0.001), and Serbia (χ^2^ = 8.040; *p* = 0.045). However, no statistically significant differences were observed in North Macedonia (χ^2^ = 13.670; *p* = 0.057) or Montenegro (χ^2^ = 2.454; *p* = 0.293).

**Figure 1:**
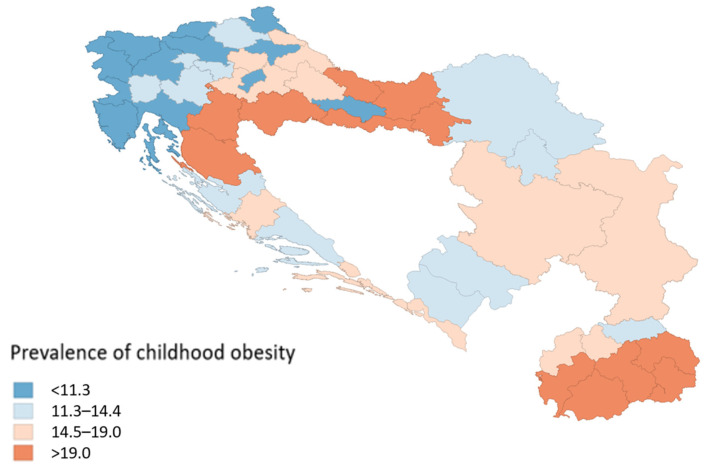
Childhood obesity prevalence across sub-national regions in Croatia, Slovenia, North Macedonia, Montenegro, and Serbia.

Figure 1 displays a map of childhood obesity prevalence across sub-national regions in five south-east European countries. A color gradient is used, with blue shades representing a lower prevalence and red shades indicating a higher prevalence. The lowest recorded prevalence of childhood obesity (7.20%) was found in the Osrednjeslovenska region of Slovenia, while the highest prevalence (33.30%) was observed in the Sisak-Moslavina region of Croatia. Information on the Sub-national Human Development Index (SHDI) and its three primary dimensions for each region is available in the Supplementary Materials.

### 3.2. Spatial Autocorrelation Analysis of Childhood Obesity Prevalence in Sub-National Regions and Its Association with Sub-National Human Development

A spatial autocorrelation analysis has been performed to analyze whether regions with similar childhood obesity prevalence values tend to be located near each other.

**Figure 2:**
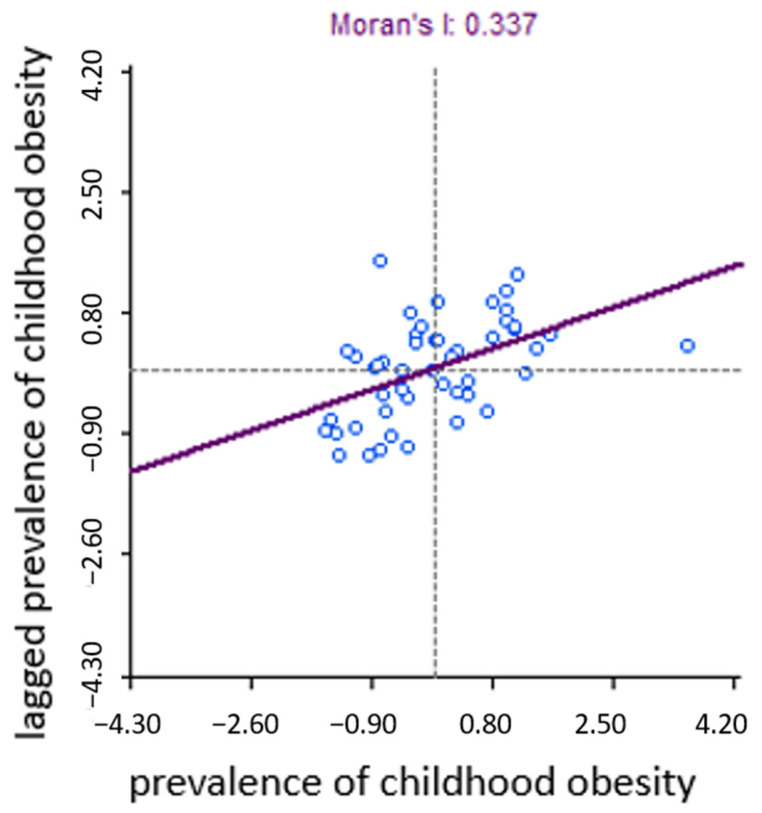
Moran scatterplot with results from a spatial autocorrelation analysis of sub-national regions and their prevalence of childhood obesity.

The Moran scatterplot in Figure 2 suggests that there is moderate clustering in childhood obesity rates (Moran’s I = 0.337) and that high-obesity regions are spatially near each other (and vice versa). The sensitivity analysis excluding regions with small sample sizes confirms moderate clustering (Moran’s I = 0.484).

**Figure 3:**
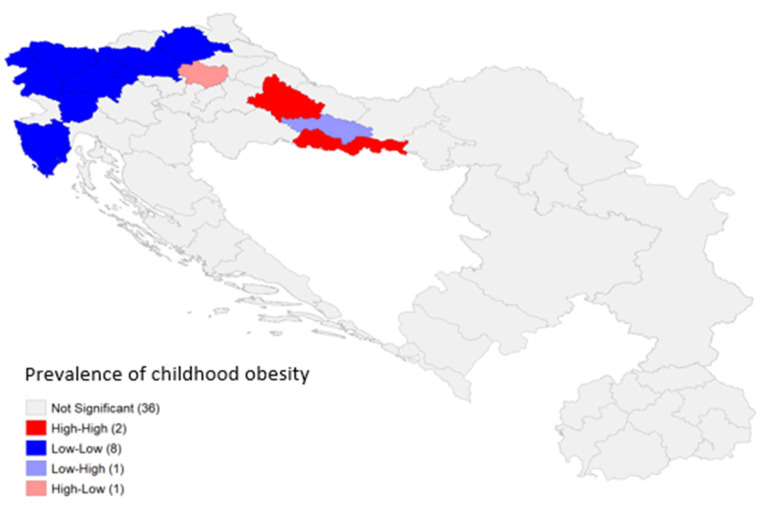
LISA cluster map (Local Indicators of Spatial Association) depicting statistically significant spatial clusters of childhood obesity prevalence.

Figure 3 shows a LISA (Local Indicators of Spatial Association) cluster map, which provides insight into the spatial autocorrelation patterns. While in most regions (36 of them) there were no statistically significant local spatial autocorrelations, some significant clusters could be found in Croatia and Slovenia. In Croatia, there were two high–high regions (i.e., regions with high childhood obesity prevalence, surrounded by other high-prevalence regions): Bjelovar-Bilogora County and Brod-Posavina County. All but five regions in Slovenia, along with the region of Istria in Croatia, could be identified as low–low regions, i.e., regions with low childhood obesity prevalence, surrounded by other low-prevalence regions. In Croatia, the Požega-Slavonia County seems to be an outlier, in the sense that it is a low–high region, i.e., it has low prevalence but is surrounded by high-prevalence neighbors. The Krapina-Zagorje County is another type of spatial outlier, as it has a high prevalence but is surrounded by low-prevalence neighbors.

The sensitivity analysis (on 44 regions, without the 4 regions in Croatia with a low sample size) showed that the clusters in Slovenia and Brod-Posavina County are still detectable, but some new low–high and high–high clusters emerged in Serbia and North Macedonia (Figure S1).

Table 2 presents estimates from an OLS model where regional childhood obesity prevalence is the dependent variable and SHDI a predictor, controlling for country fixed effects across five countries. In the OLS regression model using the full sample (n = 48), SHDI is strongly and negatively associated with childhood obesity prevalence (β = −90.7, *p* < 0.001), and this result is robust to heteroskedasticity-consistent and country-clustered standard errors. The sensitivity analysis where regions with small sample sizes were excluded (n = 44) yields nearly identical estimates (β = −87.2, *p* < 0.001), indicating that the results are not driven by potentially unreliable observations. Spatial diagnostics provide no evidence of residual spatial autocorrelation in either sample (Moran’s I *p*-values > 0.65), and Lagrange Multiplier tests do not support spatial lag or spatial error dependence. Together, these findings suggest that the full sample OLS regression model with SHDI as a predictor and regional childhood obesity prevalence as a dependent variable, with country-fixed effects, is an appropriate specification and that the inverse association between sub-national human development and childhood obesity prevalence is robust to sample restrictions and not driven by spatial dependence.

## 4. Discussion

This study examines the associations between childhood obesity and socioeconomic factors at the sub-national regional level in two high-income countries (Croatia and Slovenia) and three upper middle-income countries (Serbia, North Macedonia, and Montenegro) [34]. Previous research primarily focused on national or cross-national data, often overlooking sub-national regional heterogeneity in childhood obesity.

### 4.1. Sub-National Regional Differences in Childhood Obesity

The study identified significant sub-national regional differences in childhood obesity within Croatia, Serbia and Slovenia, but not in North Macedonia or Montenegro. These differences could stem from socioeconomic or cultural diversity within and between sub-national regions [35], given the general agreement that obesity is a multifactorial disease, strongly influenced by social, economic, and cultural determinants. However, further, more comprehensive research should be implemented to discuss this complexity within the context of the investigated countries. Additionally, variations in the effectiveness of public policy implementation at the national and sub-national levels could hypothetically contribute to these discrepancies [36]. Despite national efforts to reduce childhood obesity and advance progress toward the global nutrition targets [37,38], significant within-country disparities remain, underscoring the need for further investigation.

While previous research identified a north–south gradient in childhood obesity prevalence across Europe [3], with higher rates in southern regions [39], this study does not confirm the uniform existence of such a gradient at the regional level within south-eastern Europe. The north–south gradient observed in other European studies seems to be a result of the significant dietary shift that has arisen in the Mediterranean region, from a traditional Mediterranean diet to a more Westernized eating pattern [39,40,41]. When observing within country variability, our results support the proposed north–south gradient for Montenegro, Serbia and Macedonia, while this was not identifiable in Croatia and Slovenia, suggesting caution when generalizing such results for the entire south-eastern region. In Croatia, this is in line with newest regional trends which suggest the highest prevalence of childhood obesity in the Pannonian region [42].

### 4.2. Spatial Clustering of Childhood Obesity and Its Association with Sub-National Human Development

When examining the regional distribution of childhood obesity, several clusters were observed. The findings reveal that in Croatia and Slovenia, regions with a higher prevalence of childhood obesity tend to be located near each other, whilst the same is true for the regions with a lower prevalence. This finding could point to shared environmental, socioeconomic, or policy factors influencing children’s health at a regional level. The importance of national public health policies in addressing childhood obesity has been increasingly recognized as a key factor for shaping the spatial patterns of childhood obesity prevalence across countries. Examples of such policies include addressing food environments, the availability of physical activity, and other determinants that focus on systemic change for improving health. However, for these interventions to be effective, policies ought to be coordinated and implemented through multi-sectoral approaches [43,44]. In the context of these findings, the observed spatial clustering could support the notion that national policy environments play a significant role in shaping regional patterns of childhood obesity, although local contextual factors may ultimately determine variations in prevalence.

With these findings in mind, future research should focus on specific policies, environmental characteristics, and socioeconomic factors of potential “hot zones” of childhood obesity. Additionally, attention should be given to the study of potential “protective” zones, clusters with lower childhood obesity prevalence, whose best practices or protective factors, if empirically confirmed, could be replicated elsewhere. A substantial opportunity for future research regarding possible opportunities for directing preventive efforts lies in the “outlier regions.” Future research studies focused on examining these regions, which border areas of higher prevalence yet maintain lower childhood obesity rates and in which neither protective nor risk factors have been clearly identified, might prove to be essential for understanding the mechanisms of resilience and vulnerability, and for informing targeted public health interventions aimed at reducing regional disparities in childhood obesity.

This study also showed regions with higher human development tend to have substantially lower rates of childhood obesity. There were a few previous studies from high-income countries (e.g., Chile, Spain, and the Netherlands) showing similar patterns [45,46,47]. On the other hand, a study from Thailand (upper middle-income country) found that childhood obesity was significantly positively associated with areas exhibiting high levels of socioeconomic environment factors [48], which may point to the idea that sub-national socioeconomic development could have a different relationship to childhood obesity, depending on the economic development of the entire country. It is also of note that regional clustering of childhood obesity prevalence was found only in the two high-income countries in this study (Croatia and Slovenia), but not in the others, which might further emphasize the importance of looking at national-level development when studying regional differences in childhood obesity. However, given the cross-sectional ecological study design, these findings should be interpreted with caution and primarily serve to inform directions for future research, rather than to directly inform the development of public health policies.

This study has several strengths and limitations. One key strength is the large sample size of 25,211 children, combined with standardized procedures for weight and height measurements across all five countries, ensuring comparability. Another strength is the sub-national regional perspective, which provides more detailed insights into the potential drivers of childhood obesity at a regional level. When interpreting the results of this study, certain limitations must be considered as well. First, obesity was assessed through BMI, which, while widely used, is not the most precise method for assessing children’s nutritional status. Variations in body composition, along with the unique characteristics of south-eastern Europeans—who are among the tallest populations globally—may lead to significant misclassifications when applying international standards. Second, the sample was designed to be nationally rather than regionally representative, leading to variations in the number of children included from each region. This may explain the exceptionally high childhood obesity prevalence observed in Croatia’s Sisak-Moslavina region, which reached 33.30%. Given that only 33 children from this region were included in the total sample, the high prevalence may be influenced by the small sample size. In addition, the number of regions and the size of the regional sub-samples vary across the countries. Also, the exclusion of biologically implausible BMI values, while necessary, may introduce bias by removing extreme cases. Differences in age group selection across countries may further impact data comparability. Additionally, the use of sub-national human development indices, derived from aggregated data, limits the ability to capture the nuanced socioeconomic effects on obesity at an individual level. Also, variability in the selection of professionals conducting anthropometric measurements may introduce inconsistencies. Finally, a cross-sectional study design based on a single measurement of exposure and outcome, does not enable the establishing of causal relationships.

## 5. Conclusions

This study demonstrates that sub-national regional differences in childhood obesity in south-east Europe do exist but are inconsistent. A strong negative association was identified between childhood obesity prevalence and sub-national socioeconomic development, which may suggest the importance of looking at regional factors beyond family-level influences.

Given the ongoing global rise in childhood obesity, these findings contribute to underscoring the necessity of addressing socioeconomic disparities through comprehensive, multisectoral, and regionally specific interventions. Policymakers should prioritize reducing inequalities in childhood obesity to mitigate its public health impact.

## Figures and Tables

**Table 1:** Dimensions of the SHDI and the indicators used to create them, modified from Smits [26].

Index	Dimension	Indicator
**SHDI**	Education index	Mean years of schooling of adults aged 25+ (MYS)
		Expected years of schooling (EYS)
	Health index	Life expectancy at birth (LE)
	Standard of living/income index	(Log of) Gross national income per capita (LGNIc)

**Table 2:** Results of an ordinary least squares regression (OLS) with sub-national human development (SHDI) as a predictor and regional childhood obesity prevalence as a dependent variable, with country-fixed effects.

Variable	Full Sample (n = 48)	Sensitivity Sample (n = 44)
**SHDI**	−90.69 ** (17.46)	−87.19 ** (17.83)
**Croatia**	0.94 (1.15)	0.45 (1.08)
Serbia	−3.99 * (1.62)	−3.67 * (1.65)
North Macedonia	−4.20 (2.33)	−3.73 (2.38)
Montenegro	−3.30 * (1.26)	−3.05 * (1.29)
**Constant**	92.19 ** (15.78)	89.03 ** (16.12)
**Moran’s I (*p*)**	−0.157 (0.919)	−0.077 (0.680)

Notes: Robust standard errors clustered at the country level in parentheses. The reference category is Slovenia. Regions with small sample sizes are excluded in the sensitivity analysis. ** *p* < 0.001, and * *p* < 0.05.

## Data Availability

Data retrieved from the Subnational HDI Database of the Global Data Lab, https://globaldatalab.org/shdi/ (accessed on 29 December 2025), version v7.0. Further inquiries can be directed to the corresponding author.

## Supplementary Materials

The following supporting information can be downloaded at: https://www.mdpi.com/article/10.3390/children13020267/s1, Table S1: Number of children included in the analysis per country/region; Table S2: SHDI and its three main dimensions in Croatia; Table S3: SHDI and its three main dimensions in Slovenia; Table S4: SHDI and its three main dimensions in North Macedonia; Table S5: SHDI and its three main dimensions in Serbia; Table S6. SHDI and its three main dimensions in Montenegro; Figure S1: LISA cluster map (Local Indicators of Spatial Association) depicting statistically significant spatial clusters of childhood obesity prevalence in the sensitivity analysis run on 44 regions (excluding regions with low sample size).

## Abbreviations

The following abbreviations are used in this manuscript:

WHO	World Health Organization
COSI	Childhood Obesity Surveillance Initiative
JRC	Joint Research Center
UNDP	United Nations Development Program
HDI	Human Development Index
SHDI	Sub-national Human Development Index
MYS	Mean years of schooling of adults aged 25+
EYS	Expected years of schooling
LE	Life expectancy at birth
LGNIc	(Log of) Gross national income per capita
BMI	Body mass index
