# Supplementary material for: Association Between Sub-National Regional Socioeconomic Status and Childhood Obesity in Five South-East European Countries: The WHO European Childhood Obesity Surveillance Initiative—COSI (2019)
Source: Children (Basel). 2026 Feb 13;13(2):267. doi: 10.3390/children13020267 (PMC12939025; doi:10.3390/children13020267)
Supplement: Supplementary file 1 [file children-13-00267-s001.zip › children-4100153-supplementary.pdf]

Table S1. Number of children included in the analysis per country/region.

Number of children included in the analysis per country/region									
**Croatia**	2835	**Montenegro**	3344	**North Macedonia**	3201	**Serbia**	2078	**Slovenia**	13753
City of Zagreb	572	Centre	1279	East	483	Belgrade	473	Gorenjska	1356
Bjelovar-Bilogora County	98	North	1056	North East	320	South and East Serbia	592	Goriska	484
Brod-Posavina County	161	South	1009	Pelagoniski	541	Sumadija and West Serbia	497	Jugovzhodna Slovenija	815
Dubrovnik-Neretva County	114			Poloski	354	Vojvodina	516	Koroska	594
Istria County	179			Skopski	430			Obalno-kraska	577
Karlovac County	123			South East	501			Osrednjeslovenska	3753
Koprivnica-Krizevc County	96			South West	399			Podravska	2519
Krapina-Zagorje County	60			Vardarski	173			Pomurska	803
Lika-Senj County	23							Posavska	674
Medimurje County	128							Primorsko-notranjska	274
Osijek-Baranja County	119							Savinjska	1551
Pozega-Slavonia County	27							Zasavska	353
Primorje-Gorski Kotar County	138								
Sibenik-Knin County	84								
Sisak-Moslavina County	33								

Split-Dalmatia County	318
Varazdin County	139
Virovitica-Podravina County	49
Vukovar-Srijem County	89
Zadar County	83
Zagreb County	202

Table S2. SHDI and its three main dimensions in Croatia.

**SHDI and its three main dimensions in Croatia**				
**Sub-national region**	SHDI	Health index	Educational index	Income index
**City of Zagreb**	0.920	0.904	0.912	0.945
**Bjelovar-Bilogora County**	0.818	0.904	0.760	0.798
**Brod-Posavina County**	0.812	0.904	0.774	0.764
**Dubrovnik-Neretva County**	0.870	0.904	0.851	0.856
**Istria County**	0.879	0.904	0.842	0.891
**Karlovac County**	0.833	0.904	0.789	0.811
**Koprivnica-Krizevc County**	0.830	0.904	0.764	0.829
**Krapina-Zagorje County**	0.819	0.904	0.773	0.787
**Lika-Senj County**	0.826	0.904	0.764	0.815
**Medimurje County**	0.839	0.904	0.786	0.832
**Osijek-Baranja County**	0.804	0.904	0.800	0.820
**Pozega-Slavonia County**	0.808	0.904	0.760	0.769
**Primorje-Gorski Kotar County**	0.888	0.904	0.872	0.889
**Sibenik-Knin County**	0.837	0.904	0.790	0.820
**Sisak-Moslavina County**	0.827	0.904	0.776	0.807
**Split-Dalmatia County**	0.853	0.904	0.844	0.815
**Varazdin County**	0.845	0.904	0.807	0.827
**Virovitica-Podravina County**	0.802	0.904	0.746	0.764
**Vukovar-Srijem County**	0.811	0.904	0.767	0.771
**Zadar County**	0.843	0.904	0.806	0.822
**Zagreb County**	0.843	0.904	0.812	0.817

Table S3. SHDI and its three main dimensions in Slovenia.

SHDI and its three main dimensions in Slovenia				
**Sub-national region**	SHDI	Health index	Educational index	Income index
**Gorenjska**	0.920	0.961	0.923	0.880
**Goriska**	0.911	0.941	0.906	0.886
**Jugovzhodna Slovenija**	0.906	0.936	0.890	0.891
**Koroska**	0.892	0.921	0.890	0.866
**Obalno-kraska**	0.921	0.946	0.915	0.902
**Osrednjeslovenska**	0.956	0.967	0.947	0.952
**Podravska**	0.903	0.929	0.911	0.869
**Pomurska**	0.877	0.920	0.874	0.838
**Posavska**	0.892	0.921	0.886	0.870
**Primorsko-notranjska**	0.895	0.938	0.899	0.852
**Savinjska**	0.907	0.935	0.900	0.886
**Zasavska**	0.871	0.929	0.887	0.802

Table S4. SHDI and its three main dimensions in North Macedonia.

SHDI and its three main dimensions in North Macedonia				
**Sub-national region**	SHDI	Health index	Educational index	Income index
**East**	0.761	0.877	0.670	0.750
**North East**	0.756	0.863	0.671	0.747
**Pelagoniski**	0.782	0.844	0.739	0.768
**Poloski**	0.767	0.850	0.684	0.775
**Skopski**	0.802	0.891	0.750	0.773
**South East**	0.736	0.830	0.638	0.752
**South West**	0.781	0.866	0.712	0.773
**Vardarski**	0.780	0.881	0.701	0.768

Table S5. SHDI and its three main dimensions in Serbia.

SHDI and its three main dimensions in Serbia				
**Sub-national region**	SHDI	Health index	Educational index	Income index
**Belgrade**	0,840	0,872	0,869	0,782
**South and East Serbia**	0,802	0,872	0,765	0,774
**Sumadija and West Serbia**	0,800	0,872	0,753	0,780
**Vojvodina**	0,804	0,872	0,766	0,778

Table S6. SHDI and its three main dimensions in Montenegro.

SHDI and its three main dimensions in Montenegro				
**Sub-national region**	SHDI	Health index	Educational index	Income index
**Centre**	0,846	0,878	0,844	0,817
**North**	0,831	0,878	0,798	0,818
**South**	0,819	0,878	0,782	0,800

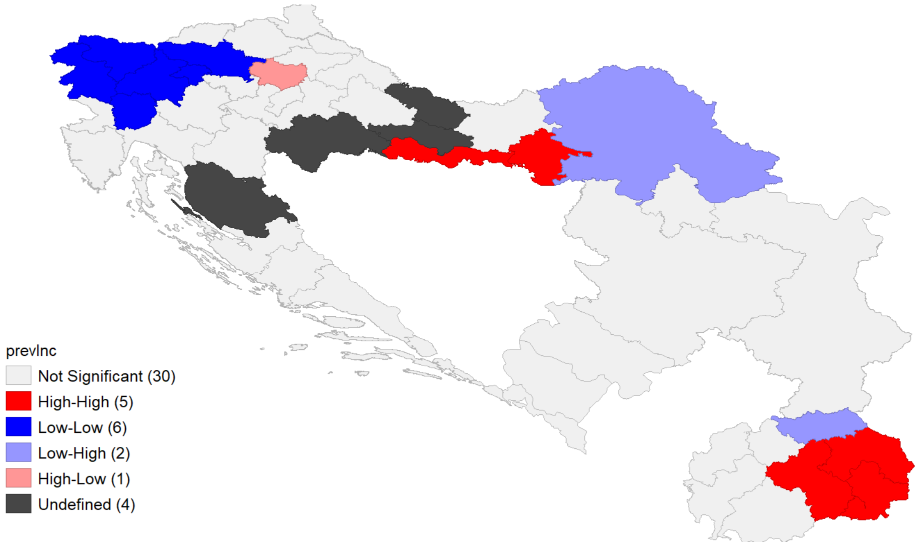

Figure S1. LISA cluster map (Local Indicators of Spatial Association) depicting statistically significant spatial clusters of childhood obesity prevalence in the sensitivity analysis run on 44 regions (excluding regions with low sample size)
